# Chemical Constituents of *Eupatorium japonicum* and Anti-Inflammatory, Cytotoxic, and Apoptotic Activities of Eupatoriopicrin on Cancer Stem Cells

**DOI:** 10.1155/2021/6610347

**Published:** 2021-05-18

**Authors:** Minh Giang Phan, Thi Thao Do, Thi Nga Nguyen, Thi Viet Huong Do, Ngoc Phuc Dong, Minh Trang Vu

**Affiliations:** ^1^Faculty of Chemistry, VNU University of Science, Vietnam National University, 19 Le Thanh Tong Street, Hanoi, Vietnam; ^2^Institute of Biotechnology, Vietnam Academy of Science and Technology, 18 Hoang Quoc Viet Road, Hanoi, Vietnam; ^3^VNU University of Education, Vietnam National University, 144 Xuan Thuy Road, Hanoi, Vietnam

## Abstract

*Eupatorium japonicum* Thunb. of the plant family Asteraceae is a popular traditional herb in Vietnam. However, its chemical constituents as well as bioactive principles have not been investigated yet. We investigated the phytochemistry of *E. japonicum* in Vietnam and isolated seventeen compounds **(1–17)** including phytosterols, terpenoids, phenolic acids, flavonoids, fatty alcohols, and fatty acids. They were structurally determined by MS and NMR analysis. Except for compounds **6** and **12**, all the other compounds were identified for the first time from *E. japonicum*. Since many sesquiterpene lactones with *α*-methylene *γ*-lactone ring are reported as anti-inflammatory and anticancer agents, eupatoriopicrin **(10)**, 1-hydroxy-8-(4,5-dihydroxytigloyloxy)eudesma-4(15),11(13)-dien-6,12-olide **(11)** were selected among the isolates for biological assays. Compound **10** was identified as the main bioactive sesquiterpene lactone of *E. japonicum* showing its potent anti-inflammatory and cytotoxic activity through inhibiting NO production and the growth of HepG2 and MCF-7 human cancer cell lines. For the first time, eupatoriopicrin **(10)** was demonstrated to strongly inhibit NTERA-2 human cancer stem cell (CSC) line *in vitro*. It is noticeable that the cytotoxicity of eupatoriopicrin against NTERA-2 cells is mediated by its apoptosis-inducing capability of **10** as demonstrated by the results of Hoechst 33342 staining, flow cytometry apoptosis analysis, and caspase-3 activity assays. The biological activities of the main bioactive constituents **1**–**7**, **10**, **12**, and **15** supported the reported anti-inflammatory and anticancer properties of extracts from *E. japonicum*.

## 1. Introduction

In Vietnam, traditional herbal remedies are considered complementary or alternative medicines in the healthcare system. However, many herbal remedies are clinically used without any scientific evidence of their efficacy and safety. Since biological activities are the links between medicinal plants and their pharmacology and medicinal uses, we have investigated the chemical constituents and biological activities of common *Eupatorium* species used in Vietnamese traditional medicine such as *E. fortunei* [1] and *E. triplinerve* [2]. Among the compounds isolated from the genus *Eupatorium*, a number of sesquiterpenoids have been proven to possess different levels of cytotoxic, anti-inflammatory, antifungal, antibacterial, and insecticidal activities [3]. *E. japonicum* Thunb. belonging to the family Asteraceae has been recorded as a medicinal plant in Vietnam, Japan, and China. Its leaves are used for the treatment of nausea, vomiting, indigestion, and diarrhea [4]. In Vietnam, the species is known under the name “Son lan” or “Yen bach Nhat” (ydvn.net) and distributed in the mountainous Sapa district of the Northwestern Lao Cai province. This perennial herb grows 1–2 m tall, blooms from August to September, and fruits from September to November. The whole plant and root of “Son lan” are used by Vietnamese people to treat cough after a cold, back pain due to cold, and irregular menstruation (ydvn.net) [5]. A very few chemical studies reported the isolation of coumarin and euponin (a guaiane-type lactone) [6] as well as the identification of pyrrolizidine alkaloids by mass spectrometry (MS) [7] from the leaves of *E. japonicum*. Forty years later, in a recent communication, 19 compounds were reported from the flowers of *E. japonicum* in China including coumarin, 2-hydroxy-2,6-dimethylbenzofuran-3(2*H*)-one, 1-(2-hydroxy-4-methylphenyl)propan-1,2-dione, subamone, (7*R*^*∗*^)-opposit-4(15)-ene-1*β*,7-diol, gentisic acid, caffeic acid, 9-hydroxythymol, protocatechuic acid, *trans*-*O*-hydroxycinnamic acid, umbelliferone, quercetin, taraxasterol, 9-acetoxythymol 3-*O*-tiglate, taraxasterol acetate, linoleic acid, 9-angeloyloxythymol, stigmasterol, and palmitic acid [7]. The present study describes the first investigation of the chemical constituents of the leaves of *E. japonicum* in Vietnam which resulted in the isolation of 17 compounds of different polarities and structural classes ranging from fatty alcohol, fatty acid, monoterpene, sesquiterpene lactone, and phytosterol to phenolic acid and flavonoid glycoside. Except for compounds **6** and **12**, all of the compounds were isolated and identified for the first time from *E. japonicum*.

Compounds derived from nature create prominent screening libraries for drug discovery. Owing to a vast reservoir of unexploited medicinal plants, the Vietnamese medicinal plants have the potential to offer compounds of various structural and stereochemical types for the screening of lead compounds [8]. The safety of ethanol extracts prepared from *E. japonicum* and *Foeniculum vulgare* was evidenced from a recent toxicity test in mice [9]. Stem extracts from *E. japonicum* suppressed lipid accumulation and inhibited the expression of adipocyte markers and coumaric acid and its methyl ester were isolated from the extracts as the bioactive components [10]. Extracts from *E. japonicum* exerted anti-inflammatory effects through the suppression of various molecular targets such as NO production, interleukin, iNOS, COX-2, MMP-9 transcription, NF-*κ*B activation, and TRIF-dependent signaling pathway of toll-like receptors [4, 11, 12]. In addition, apoptotic effects through the ROS-induced ATF4 and CHOP expression [4] and antimetastatic effects through inhibiting cell migration, invasion, and adhesion of MDA-MB-231 human breast cancer cells in dose-dependent manners were observed for the extracts [13]. The major compounds responsible for these biological activities of *E. japonicum* have not been revealed yet. Since many sesquiterpene lactones with *α*-methylene *γ*-lactone ring are reported to regulate anti-inflammatory and anticancer targets [14, 15] in order to validate the biological effects of *E. japonicum* extracts in relation to its chemical constituents eupatoriopicrin **(10)**, a germacranolide and 1-hydroxy-8-(4,5-dihydroxytigloyloxy)eudesma-4(15),11(13)-dien-6,12-olide **(11)**, an eudesmanolide were selected for the study of NO-inhibitory activity and cytotoxicity against human cancer stem cells (CSCs). Being disclosed as cytotoxic, the mechanism of cell death through apoptosis induction on CSCs would be examined using Hoechst 33342 staining, flow cytometry apoptosis analysis, and caspase-3 activity assays on NTERA-2 pluripotent human embryonal carcinoma cell line.

Nitric oxide (NO) is a short-lived free radical derived from L-arginine by nitric oxide synthase (NOS) that mediates diverse functions by acting on various cells through interactions with different molecular targets. The excessive production of NO in prolonged inflammation can cause cellular and tissue damage. The adverse effect of this inflammation can be prevented by the intervention of anti-inflammatory agents. Compounds able to inhibit the production of NO or other proinflammatory molecules are considered potential anti-inflammatory agents. CSCs are the cells that have the ability to self-renew, differentiate into defined progenies, initiate, and maintain tumor growth [16]. Despite being a small percentage in tumor cells, CSCs are present in most tissues including breast, brain, lung, head and neck, prostates, testis, ovary, esophagus, colon, and liver and play a role in cancer metastasis and therapeutic resistance, both of which are the major causes of cancer mortality and cancer recurrence [17]. There is evidence that the current chemotherapy for the treatment of cancer is insufficient in eliminating CSCs from a number of cancer types. The lingering CSCs are able to form new, fully developed tumors from a small number of cells or even a single cell. Therefore, recent studies target the discovery of natural drugs against CSCs involved in tumor development for different cancers [18]. Apoptosis is a highly regulated cell death process and occurs both during normal development and under certain pathological conditions. Apoptosis plays a crucial role in maintaining tissue homeostasis by the selective elimination of excessive cells. The evasion of apoptosis is an essential hallmark of cancer; therefore, killing cancer cells through the induction of apoptosis is a valuable tool for cancer treatment.

## 2. Materials and Methods

### 2.1. General Experimental Procedures

IR spectra were taken on an Affinity-1S FT-IR spectrometer. Electron-spray ionization-mass spectrometry (ESI-MS) spectra were measured on an Agilent 1100 LC-MSD-Trap-SL system (Agilent Technologies, USA) or Thermo Fisher Scientific LTQ Orbitrap XL^MS^ mass spectrometer in CH_3_OH solution. ^1^H-NMR (SF 500.20 MHz), ^13^C-NMR (SF 125.13 MHz), and DEPT spectra were recorded in CDCl_3_ or CD_3_OD using a Bruker Avance 500 NMR spectrometer. The chemical shifts are expressed in ppm relative to tetramethylsilane (TMS) as an internal standard. Silica gel 40–63 *μ*m or 15–40 *μ*m, reversed-phase C-18 silica gel (RP-18) 40–63 *μ*m (Merck, Germany), and highly porous Diaion HP-20 (Mitsubishi, Japan) were used for column chromatography (CC). Precoated silica gel Merck 60 F_254_ aluminum plates were used for thin-layer chromatography (TLC). Lipopolysaccharide (LPS) from *Escherichia coli* was purchased from Sigma Chemical Co. (St. Louis, MO, USA). Dulbecco's Modified Eagle's Medium (DMEM) and fetal bovine serum (FBS) were purchased from Life Technologies, Inc. (Gaithersburg, MD, USA). Sodium nitrite, sulfanilamide, *N*-1-napthylethylenediamine dihydrochloride, dimethyl sulfoxide (DMSO), sulforhodamine B (SRB), 3-(4,5-dimethylthiazol-2-yl)-2,5-diphenyltetrazolium bromide (MTT), and 4-(2-hydroxyethyl)-1-piperazineethanesulfonic acid (HEPES) were obtained from Sigma Chemical Co. (St. Louis, MO, USA). All cell lines were a kind gift from Professor Domenico Delfino, Perugia University, Italy, while the pluripotent human embryonal carcinoma cell line (NTERA-2) was provided by Dr. P Wongtrakoongate, Mahidol University, Thailand.

### 2.2. Plant Materials

The leaves of *E. japonicum* were collected in Lao Cai province, Vietnam, in September 2016. The plant material was taxonomically identified by Dr. Nguyen Thi Kim Thanh, Faculty of Biology, VNU University of Science, Vietnam National University, Hanoi, and deposited at the same institution (voucher sample EJ-916).

### 2.3. Extraction and Isolation of Compounds 1–17

The dried powdered leaves (2.0 kg) of *E. japonicum* were extracted with MeOH at room temperature (3 times, each for 7 days). The combined MeOH extract was concentrated under reduced pressure, and the residue was successively partitioned between water and organic solvents of increasing polarities to give *n*-hexane- (120 g) and CH_2_Cl_2_-soluble fractions (12 g). The water phase was concentrated under reduced pressure to give a water-soluble fraction. The *n*-hexane- and CH_2_Cl_2_-soluble fractions were combined and a part (66.7 g) was separated by silica gel CC eluting with *n*-hexane-acetone 9 : 1, 6 : 1, 5 : 1, 4 : 1, and 3 : 1 (v/v) to give eight fractions. Fraction 1 (3.09 g) was separated by silica gel CC eluting with *n*-hexane-acetone 100 : 1, 50 : 1, and 1 : 1 to give a mixture of **1** and **2** (5.0 mg) and a mixture of **3** and **4** (5.0 mg). A mixture of **5** and **6** (20 mg) was crystallized from fraction 3 (1.46 g). Fraction 5 (4.4 g) was separated by silica gel CC eluting with *n*-hexane-EtOAc 15 : 1, 12 : 1, 9 : 1, 6 : 1, 3 : 1, and 1 : 1 to give five fractions 5.1–5.2. Fraction 5.1 (1.2 g) was separated by silica gel CC eluting with *n*-hexane-EtOAc 90 : 1, 49 : 1, 25 : 1, 15 : 1, and 12 : 1 to give a mixture of fatty alcohols (113.1 mg) and **5** (105 mg). Fraction 5.5 (1.2 g) was separated by silica gel CC eluting with CH_2_Cl_2_-MeOH 30 : 1, 25 : 1, 20 : 1, 15 : 1, and 9 : 1 to give **7** (7.5 mg). Fraction 6 (22.8 g) was separated by silica gel CC eluting with *n*-hexane-EtOAc 15 : 1, 12 : 1, 9 : 1, and 6 : 1 to give five fractions 6.1–6.5. Fraction 6.3 (0.8 g) was separated by silica gel CC eluting with *n*-hexane-acetone 15 : 1 to give **8** (5.7 mg). Fraction 6.5 (4.7 g) was separated by repeated silica gel CC using CH_2_Cl_2_-acetone 15 : 1, 12 : 1, 9 : 1, 6 : 1, 3 : 1, and 1 : 1 to give a glycerol ester (4.5 mg). Fraction 7 (18.6 g) was crystallized with acetone to give **9** (86.1 mg). The residue was separated by silica gel CC eluting with CH_2_Cl_2_-MeOH 90 : 1, 49 : 1, 40 : 1, and 30 : 1 to give five fractions. Fractions 7.3 and 7.4 gave an insoluble solid in CH_2_Cl_2_. The solid was separated by silica gel CC eluting with CH_2_Cl_2_-acetone 9 : 1 to give **10** (29 mg). Fraction 7.3 was separated by silica gel CC eluting with CH_2_Cl_2_-acetone 9 : 1, 6 : 1, 3 : 1, and 1 : 1 to give **11** (6.7 mg). The water-soluble fraction was passed through a Diaion HP-20 column eluting with MeOH–H_2_O 20, 40, 60, and 100% MeOH to give four corresponding fractions. The 20%-MeOH fraction (5 g) was separated by silica gel CC eluting with EtOAc-MeOH 25 : 1, 15 : 1, 9 : 1, 6 : 1, 3 : 1, and 1 : 1 to give five fractions. Fraction 1 was purified by silica gel CC eluting with CH_2_Cl_2_-acetone 30 : 1, 25 : 1, 20 : 1, 15 : 1, 10 : 1, 5 : 1, and 3 : 1 to give **12** (2.0 mg). Fraction 2 was crystallized with MeOH to give **13** (20 mg). The 40%-MeOH fraction (10 g) was separated by silica gel CC eluting with CH_2_Cl_2_-MeOH 50 : 1, 25 : 1, 15 : 1, 12 : 1, 9 : 1, 6 : 1, and 3 : 1 to give ten fractions. Fraction 2 was separated by repeated silica gel CC eluting with CH_2_Cl_2_-MeOH 90 : 1, 80 : 1, 70 : 1, 60 : 1, 50 : 1, 40 : 1, 30 : 1, 25 : 1, 20 : 1, 15 : 1, 12 : 1, 9 : 1, 5 : 1, and 3 : 1 and then CH_2_Cl-acetone 25 : 1, 20 : 1, 15 : 1, 12 : 1, 9 : 1, and 5 : 1 to give **14** (2.0 mg). Fraction 7 (0.75 g) was separated by silica gel CC eluting with EtOAc-MeOH 25 : 1, 20 : 1, 15 : 1, 12 : 1; 9 : 1; 5 : 1, 3 : 1, and 1 : 1 to give **15** (85.8 mg). The 60%-MeOH fraction (17.9 g) was separated by silica gel CC eluting with CH_2_Cl_2_–MeOH 35 : 1, 25 : 1, 15 : 1, 12 : 1, and 9 : 1 to give **10** (69.6 mg) and **16** (50.4 mg). The 100%-MeOH fraction (0.83 g) was separated by silica gel CC eluting with CH_2_Cl_2_–MeOH 50 : 1, 25 : 1, 15 : 1, 12 : 1, 9 : 1, 6 : 1, and 3 : 1 to give five fractions. Fraction 2 was separated by silica gel CC eluting with *n*-hexane-EtOAc 70 : 1, 50 : 1, 25 : 1, 15 : 1, 12 : 1, 9 : 1, 7 : 1, 5 : 1, 3 : 1, and 1 : 1 to yield **17** (2.0 mg). Fraction 4 was separated by silica gel CC eluting with *n*-hexane-EtOAc 70 : 1, 50 : 1, 25 : 1, 15 : 1, 12 : 1, 9 : 1, 7 : 1, 5 : 1, and 3 : 1 to yield a mixture of **3** and **4** (50.4 mg).

### 2.4. Cell Culture

All cell lines were seeded and cultured in DMEM medium with 2 mM L-glutamine, 10 mM HEPES, 1.0 mM sodium pyruvate, and 10% FBS in the incubator at 37°C and 5% CO_2_. Cells were subcultured every 3 days at a 1 : 3 ratio.

### 2.5. Nitric Oxide Assay

RAW 264.7 cells were seeded to a 96-well plate at a concentration of 2 × 10^5^ cells/well. After incubation for 24 h, the cultured medium was refreshed by DMEM without FBS. Then, the cells were treated with samples at different concentrations in the presence of LPS (1 *µ*g/mL) for 24 h. N^G^-Methyl-L-arginine acetate (L-NMMA) (Sigma) was used as the positive control and the cells treated with a diluted solution of DMSO (1.0%) were used as the negative control. Nitrite (NO_2_^_^), an indicator of NO production, was determined by the Griess reagent system (Promega Corporation, WI, USA). Briefly, 100 *µ*L medium was mixed with 50 *μ*L 1% (w/v) sulfanilamide in 5% (v/v) phosphoric acid and 50 *μ*L 0.1% (w/v) *N*-1-naphthylethylenediamine dihydrochloride in a 96-well plate. The plate was then incubated at room temperature for 10 min. The optical density (OD) was measured at 540 nm by using a microplate reader (Biotek, Winooski, VT, USA). The concentration of nitrite in each well was calculated by using the NaNO_2_ standard curve. The ability of NO inhibition of a sample was calculated by the following formula: (%) inhibition = 100%—(concentration of NO_sample_/concentration of NO_negative control_) × 100. The value of IC_50_ (the half-maximal inhibitory concentration) was determined by using the Table Curve 2Dv4 software (Systat Software Inc., San Jose, CA, USA) [19].

### 2.6. Cell Viability MTT Assay

The proliferation of cells treated with samples was determined by MTT assay [20]. 10 *µ*L MTT (5 mg/mL) was added to the wells containing 5 × 10^3^ RAW 264.7 cells treated with samples and then incubated at 37°C for 4 h. The purple formazan crystals formed by metabolically active cells were diluted by adding 100 *µ*L DMSO (100%) to each well after removing the medium. The OD was measured by using a microplate reader (BioTek, Winooski, VT, USA) at 540 nm. The percentage of cell viability was determined by the following formula: (%) cell viability = (OD _(sample)_—OD _(blank)_)/(OD _(negative control)_—OD _(blank)_) × 100%. All analyses were performed in triplicate and data were reported as mean ± SEM (standard error of the mean).

### 2.7. SRB Colorimetric Assay

SRB assay was used for determining the cytotoxic activity of tested samples in a 96-well plate according to the method of Skehan et al. [21]. The test evaluates the cell density based on the measurement of the total cellular protein content stained with sulforhodamine B (SRB). Briefly, trypsinized cells were seeded in a 96-well plate and incubated with sample for 48 h. The cell-containing wells treated with diluted solution serve as the negative control. After a period of incubation time, the cells were fixed with TCA for 1 h and then stained with SRB for 30 min at 37°C. The plates were washed three times with acetic acid to remove all nonstaining dye and allowed to dry at room temperature. SRB staining protein in cells was dissolved in 10 mM unbuffered Tris base and the plate was gently shaken for 10 min at room temperature. The OD at the wavelength of 540 nm was measured by using an ELISA plate reader (BioTek, Winooski, VT, USA)). The inhibition percentage of cell growth in the presence of the treated sample is determined by the following formula: (%) inhibition = 100%—(OD_(sample)_—OD_(day 0)_)/(OD_(negative control)_—OD_(day 0)_) × 100. The test was triplicated to ensure accuracy. Ellipticine was used as the positive control. The IC_50_ value was determined by using the TableCurve 2Dv4 computer software.

### 2.8. Hoechst 33342 Staining

The morphology of cell nuclei treated with samples was determined through Hoechst 33342 staining (Allen et al. [22]). NTERA-2 cells grew stable in a 6-well plate for 24 h before being treated with samples, the reference (camptothecin) or the negative control (DMSO 1%). After 48 h of treatment, cells were fixed with formaldehyde 4% for 30 min and then washed with phosphate-buffered saline (PBS) before staining with Hoechst 33342 (0.5 *µ*g/mL) for 10 min. The morphological change of nuclei was observed under a fluorescence microscope at 350⁄461 nm (excitation/emission). The apoptotic cells have brighter nuclei or fragmented chromatin.

### 2.9. Flow Cytometry Apoptosis Analysis

The flow cytometry protocol was performed using Kit Annexin V and PI/dead cell apoptosis® (Invitrogen, Thermo Fisher Scientific) according to Ngo et al. [23]. Basically, cells (1 × 10^6^ cells) treated with samples were collected after 48 h incubation and washed with PBS before staining with Annexin V-FITC for 15 min. The cells were continuously stained with propidium iodide (PI) (5 *μ*L) in Annexin V binding buffer. Total 10.000 cells for each sample were analyzed using Attune NxT flow cytometer (Thermo Fisher Scientific, USA). The unaffected cells were PI-negative and Annexin V-negative, while the apoptotic cells were Annexin V-positive, and the necrotic cells were only positive for PI.

### 2.10. Caspase-3 Activity Assay

The experiment was performed using the caspase-3 colorimetric assay kit (BioVision Inc., USA). Briefly, cells were grown in a 6-well plate for 24 h before they were treated with samples and incubated in a high-humidity CO_2_ incubator at 37°C for further 48 h. Cells that were treated with DMSO 10% were considered the negative control. After the treatment period, cells were detached with Trypsin-EDTA and then washed with PBS. The cell pellet was lysed with 50 *µ*L lysis buffer from the kit on ice for 10 min. The cell lysis solution was then reacted with 10 mM DTT and 4 mM DEVD-pNA and incubated at 37°C for 2 h. Samples were read at 405 nm by using a microplate reader to determine the OD. Fold-change in caspase-3 activity of the sample was measured by comparing with the OD of the negative control [24].

### 2.11. Statistical Analysis

The data were expressed as the mean of three replicate determinations ± standard deviation (SD). Statistical comparisons were made with Student's test; *p* values < 0.05 were considered significantly different.

## 3. Results and Discussion

### 3.1. Isolation and MS/NMR Elucidation of Compounds 1–17

**Figure 1:**
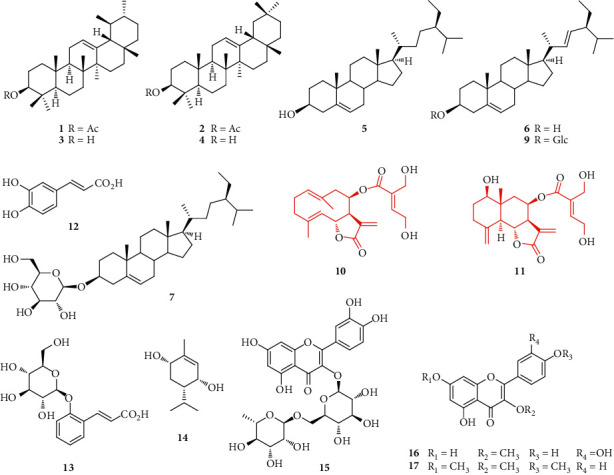
Structures of compounds **1**–**17**.

The MeOH extract from the leaves of *E. japonicum* afforded 17 compounds, a glycerol ester, and a mixture of fatty alcohols on successive liquid-liquid fractionation and repeated chromatographic separation on silica gel, RP-18, or Diaion HP-20. The structures of the compounds were identified as *α*-amyrin acetate (**1**) [25], *ß*-amyrin acetate (**2**) [25], *α*-amyrin (**3**) [25], *ß*-amyrin (**4**) [25], *ß*-sitosterol (**5**) [25], stigmasterol (**6**) [25], *β*-sitosterol 3-*O*-*β*-D-glucopyranoside (daucosterol) (**7**) [2, 3], behenic acid (**8**) [26], stigmasterol 3-*O*-*β*-D-glucopyranoside (**9**) [25], eupatoriopicrin (**10**) [27, 28], 1-hydroxy-8-(4,5-dihydroxytigloyloxy)eudesma-4(15),11(13)-dien-6,12-olide (**11**) [29, 30], caffeic acid (**12**) [31], (2*E*)-3-[2-(*β*-D-glucopyranosyloxy)phenyl]-prop-2-en-oic acid (**13**) [3], *p*-menth-1-ene-3,6-diol (**14**) [32], quercetin-3-*O*-rutinoside (rutin) (**15**) [33], quercetin 3-*O*-methyl ether (**16**) [34], and kaempferol 3,7,4′-trimethylether (**17**) [35] which were determined by comparing their spectroscopic data (MS, IR, ^1^H-NMR, and ^13^C-NMR) with reported literature values. Compounds **1**–**5**, **7**–**11**, and **13**–**17** were isolated for the first time from *E. japonicum* (Figure 1).

### 3.2. NO Production Inhibitory Activity of Compounds 10 and 11

Nitric oxide (NO) participates in various responses of host resistance to various pathogens and joins to adjust various aspects of life such as vascular integrity, maintains hemostasis, or modulates neural activity [36, 37]. The overproduction of NO induced pathological problems related to acute and chronic inflammatory, apoptosis and necrosis, or neurodegenerative diseases [38, 39]. RAW 264.7 cells activated by LPS produce large amounts of NO. Therefore, LPS-induced production of NO in murine macrophage RAW 264.7 cells was used for screening useful compounds for the development of new anti-inflammatory agents. The inhibition of NO production of compounds **10** and **11** is shown in Table 1. Compound **10** strongly inhibited NO production in LPS-stimulated RAW 264.7 cells in a dose-dependent manner with an IC_50_ value of 7.53 ± 0.28 *µ*g/mL. The positive control L-NMMA exhibited its IC_50_ value of 8.21 ± 0.84 *µ*g/mL. Cytotoxicity of **10** was observed at the active concentrations; the cell viability was 43.27% at 4 *µ*g/mL (NO inhibition: 93.83%), but up to 85.64% at 0.8 *µ*g/mL (NO inhibition: 85.02%) (Table 1). Therefore, the dose must be adjusted to a suitable level to balance drug safety and effective suppression of inflammatory responses. Previously, **10** was reported to show strong anti-inflammatory activity through the inhibition of IL-8 and TNF-*α* release in lipopolysaccharide- (LPS-) stimulated human neutrophils [14]. Altogether, the results demonstrated the promise of **10** as a lead compound towards inflammation. Compound **11** did not display potent suppression on NO production at all concentrations tested (0.4, 8, 20, and 100 *µ*g/mL). The highest inhibition of NO production was 48.02% at 100 *µ*g/mL with the cell viability reaching 95.25%. Among the isolates **1**–**17**, the inhibition of NO generation, inflammatory cytokines, tumor necrosis factor-*α*, or COX-2 enzyme was reported for triterpenes **1**–**4** [40–42], phytosterols **5**–**7** [43], and phenolic compounds **12** and **15** [44, 45]. The other compounds are not the major ones in the extracts and may not affect the overall anti-inflammatory property of the extracts.

### 3.3. Cytotoxic Activity of Compounds 10 and 11

**Figure 2:**
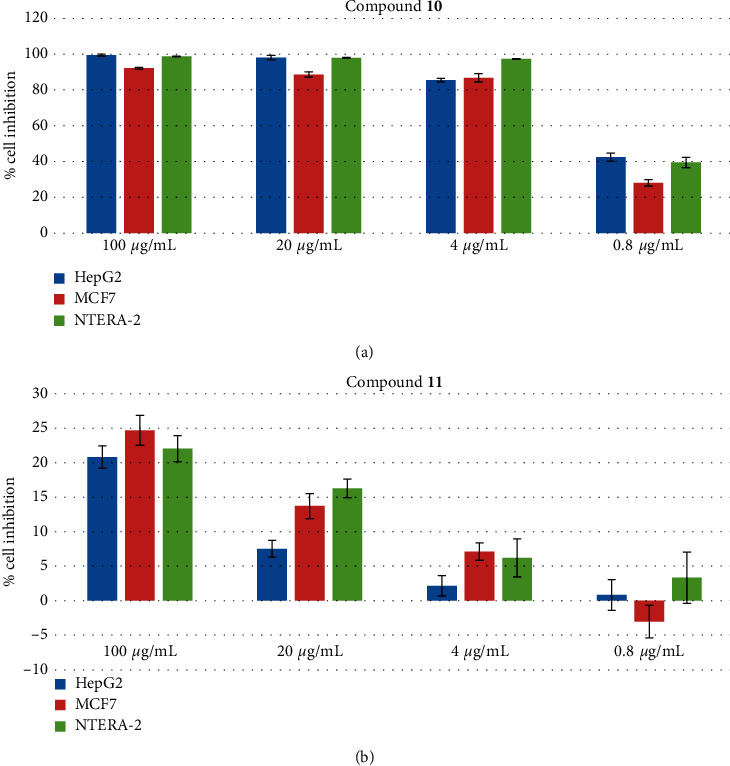
Effectiveness of compounds **10** and **11** on different cell lines, including CSCs. Cultured cells (1 × 10^4^ cells/well) were treated with different concentrations of compounds ranging from 0.8 *µ*g/mL to 100 *µ*g/mL. DMSO 1% served as the negative control. Each value represents the mean ± SEM.

NTERA-2 is an embryonal carcinoma cell line, which expresses a gene profile related to pluripotency of stem cells such as OCT3/4 and SSEA-4. The cell line presents a lot of early embryo antigens that could be used to identify undifferentiated human embryonic stem cell (hESC) [46, 47]. In addition, this cell line is easy to culture and grows quickly in a medium without any additional growth supplements except FBS. Thus, this cell line was widely used as a model for CSC research *in vitro* and in discovering novel anticancer compounds against CSCs, a research area of great interest nowadays. The cytotoxicity of compounds **10** and **11** on human breast cancer cell line (MCF7), human hepatocellular carcinoma (HepG2), and pluripotent human embryonal carcinoma cell line (NTERA-2) was accessed using SRB assay. As shown in Figure 2 and Table 2, eupatoriopicrin (**10**) showed strong cytotoxic activity on all tested cell lines with IC_50_ values of 1.22 ± 0.10 *µ*g/mL, 0.94 ± 0.12 *µ*g/mL, and 0.88 ± 0.05 *µ*g/mL, respectively. Compound **11** did not show cytotoxic effects on HepG2, MCF-7, and NTERA-2 cell lines at the concentrations tested. Hitherto compound **10** expressed its cytotoxic activity against KB, HeLa, HL-60, EN19, EAT, P388, FIO 26, and L5178Y cell lines [28, 48, 49]. The structural requisite for the potent cytotoxic activity of **10** may be associated with its *α*-methylene-*γ*-butyrolactone moiety [50]. The oncolytic activity was also demonstrated in an *in vivo* study showing the tumor growth delay after administration of eupatoriopicrin [51, 52]. The present study is the first report on the cytotoxicity of **10** against human CSCs (NTERA-2) as well as human cancer cell lines HepG2 and MCF-7. Glutathione (GSH) depletion is a central signaling event that regulates the activation of cell death pathways [53]. An *in vitro* study by Woerdenbag et al. showed that significant GSH reduction began to occur with concentrations of eupatoriopicrin ≥1 *μ*g/mL in FIO 26 cells [52]. Eupatoriopicrin may react with the sulfhydryl group of cellular GSH to form a Michael adduct and its cytotoxicity may increase by the action of GSH depletion. Two other distinct pathways of cell death are apoptosis and necrosis. To investigate the possibility of apoptosis in the cytotoxicity of **10**, we assayed further the apoptosis-inducing activity of **10** in NTERA-2 cells.

### 3.4. Apoptosis-Inducing Activity of Compound 10

**Figure 3:**
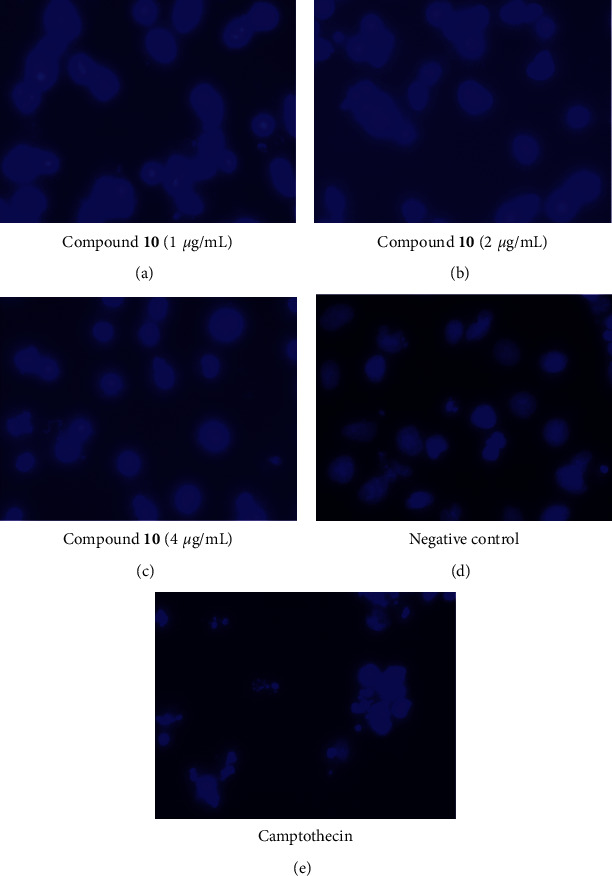
Nuclear condensation and fragmentation effects of compound **10** on NTERA cells at different concentrations as 1 *µ*g/mL, 2 *µ*g/mL, and 4 *µ*g/mL using Hoechst 33342 staining. The cells were at 48 h of incubation and observed with Zeiss Vert A1 inverted microscope (100x).

One of the promising strategies for the identification of cytotoxicity of anticancer compounds is the induction of apoptosis of cancer cells. Based on the strong cytotoxicity of compound **10** on NTERA-2 CSC line, **10** was further evaluated for its apoptosis-inducing activity to better understand the possible apoptosis pathway. We examined the possible induction of apoptotic cell death by **10** using flow cytometry analysis and activation of caspase-3 in CSCs. Thus, NTERA-2 cancer cells were treated with **10** and stained with Hoechst 33342. Using this nuclear staining dye, apoptotic cells with condensed and fragmented nuclei were detected, while most negative control cells showed nuclei with homogeneously staining morphology (Figure 3). The experiment exhibited that compound **10** could induce apoptosis on NTERA-2 cells in a dose-dependent manner (Table 3), resulting in the inhibition of the proliferation of NTERA-2 cells.

**Figure 4:**
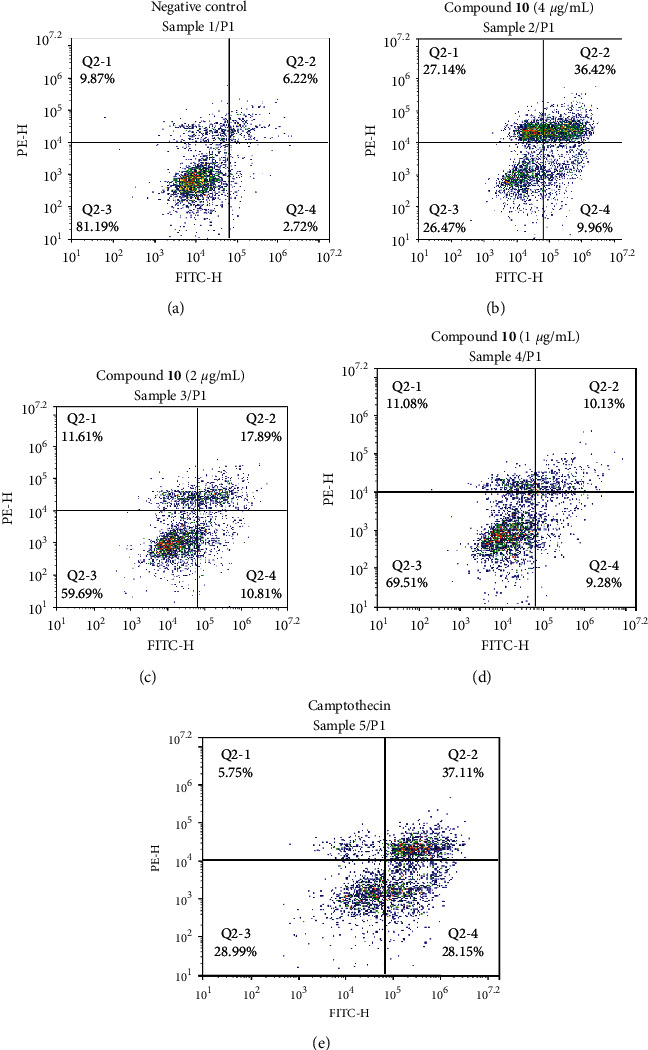
Flow cytometry analysis of apoptosis-inducing activities of compound **10** at different concentrations ranging as 1 *µ*g/mL, 2 *µ*g/mL, and 4 *µ*g/mL on NTERA-2 cells after 48 h incubation, using NovoCyte flow cytometry system and NovoExpress software (ACEA Bioscience, Inc.).

The flow cytometric analysis confirmed the apoptosis-inducing activity of compound **10**. As shown in Figure 4, the number of apoptotic cells, which was positive with Annexin V, increased along with raising concentrations of **10**. At the highest concentration, 4 *µ*g/mL **10** could induce up to 44.38% of apoptotic cells, while the anticancer drug camptothecin induced 65.26% of apoptotic cells. However, compound **10** also caused necrosis at this concentration, which was 27.14%. The necrosis was reduced when cells were exposed to lower concentrations of **10** such as 1.0 and 2.0 *µ*g/mL; meanwhile, the apoptosis-inducing activity of **10** was maintained at 19.41 and 28.70%, respectively. The results indicated that the induction of apoptosis could be a mechanism of the cytotoxicity of **10** against NTERA-2 cancer cells.

**Figure 5:**
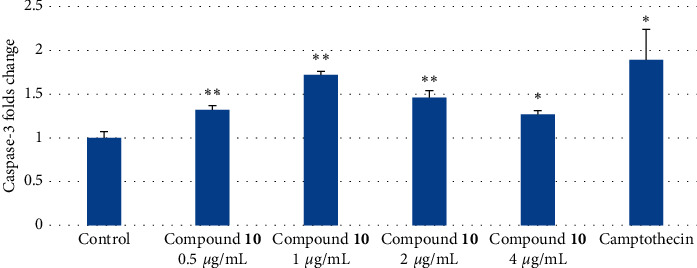
Caspase-3 inducible effects of compound **10** at different concentrations ranging from 0.5 *µ*g/mL to 4 *µ*g/mL on NTERA-2 cells after 48 h incubation. Diluted solution DMSO 1% served as the negative control. Each value represents the mean ± SEM. ^*∗∗*^*p* < 0.01 and ^*∗*^*p* < 0.05 compared to the negative control.

In order to confirm the apoptosis-inducing activity of compound **10** towards CSCs, caspase-3 activity was measured. The initiator phase of apoptosis is characterized by initiator caspase (cysteine-dependent aspartate-directed protease) activation (Franco and Cidlowski). Caspase-3 is one of the key enzymes in apoptosis and is activated in both the intracellular and extracellular apoptotic pathways [54, 55]. This enzyme specifically activates the caspase-activated DNAse which causes chromosomal degradation and condensation in the nucleus, which are important feature characteristics of apoptosis [56]. Compound **10** significantly induced caspase-3 production at all tested concentrations (*p* < 0.05) (Figure 5). Thus, compound **10** induced apoptosis in NTERA-2 cancer cells by the activation of caspase-3. However, the strongest activity was observed at the concentration of 1.0 *µ*g/mL (*p* < 0.01). Higher or lower concentrations also mediated caspase-3 production but the induction was not as strong as that at 1.0 *µ*g/mL. These results indicated that this concentration might be the optimized concentration of **10** to induce apoptosis in the NTERA-2 CSC line through the activation of caspace-3.

Throughout, **10** expressed the potential activity against CSCs by apoptosis and/or necrosis induction. A challenge of cancer therapy is multidrug resistant, which is closely related to CSCs [57]. Multidrug resistance of CSCs is caused by multiple reasons such as overexpression of ATP-binding cassette (ABC) transporters, high level of multidrug resistance (MDR), or detoxification proteins. The high survival capacity of CSCs under the treatment of drugs is caused by induced apoptosis resistance through changing a series of apoptosis mechanisms as downregulation p53 gene, miRNA action, or inhibiting apoptotic proteins [58–60]. Therefore, drugs as **10** that reduce survival rates of CSCs and induce apoptosis attract considerable attention.

## 4. Conclusions

In this study, 15 compounds out of 17 isolates were isolated for the first time from *E. japonicum*. Belonging to the privileged group of anti-inflammatory and anticancer structures, two sesquiterpene lactones **10** and **11** were selected for biological assays. Eupatoriopicrin **(10)** was found to strongly inhibit NO production, MCF-7, HepG2, and NTERA-2 cancer cell lines; meanwhile, compound **11** was not active. The cytotoxicity on the NTERA-2 cancer cell line is related to significant apoptosis induction by the action of **10** in the concentration- and caspase-dependent manners. The importance of the findings is that the study provides a chemical and biological basis for developing new approaches to explore therapeutic values of extracts or herbal medicines containing the herb *E. japonicum*. In addition, anti-inflammatory and anticancer botanical drugs enriched with bioactive constituents can be formulated and prepared from *E. japonicum*. Eupatoriopicrin **(10)** is also exposed in the study as a lead compound targeting inflammation or cancer diseases. Further projects on the synthesis of chemical derivatives or analogs of **10** addressing optimization of drug safety and effectiveness in combating inflammatory or cancer diseases are underway.

## Figures and Tables

**Table 1:** NO-inhibitory activities of compounds **10** and **11**.

Concentration (*µ*g/mL)	**10**		**11**		**L-NMMA**	
	% inhibition	% cell survival	% inhibition	% cell survival	% inhibition	% cell survival
100	—	—	48.02	95.25	92.07	89.43
20	—	—	10.44	96.65	72.78	92.93
4	93.83	43.27	3.08	—	30.21	—
0.8	85.02	86.54	−4.85	—	15.77	—
0.16	25.11	96.58	—	—	—	—
0.032	12.61	99.82	—	—	—	—
IC_50_	7.53 ± 0.28	—	>100	—	8.21 ± 0.84	—

**Table 2:** Cytotoxic activities of compounds **10** and **11** on different cell lines.

Samples	Values of IC_50_ (*µ*g/mL)		
	HepG2	MCF-7	NTERA-2
Compound **10**	0.94 ± 0.12	1.22 ± 0.10	0.88 ± 0.05
Compound **11**	>100	>100	>100
Ellipticine	0.46 ± 0.05	0.36 ± 0.04	0.49 ± 0.04

**Table 3:** Percentage of apoptosis cells induced by compound **10** on NTERA-2 cells.

Concentration (*µ*g/mL)	% apoptosis cells		
	**10**	Camptothecin (5 *µ*M)	Negative control
4	47.29 ± 5.43	84.85 ± 5.38	4.14 ± 1.43
2	34.76 ± 3.53	—	—
1	29.65 ± 5.58	—	—

## Data Availability

The data used to support the findings of this study are available from the corresponding author upon request.

## Supplementary Materials

Supplementary Materials Experimental details and MS and NMR spectra of compounds **1**–**17** are freely available along with the manuscript as supplementary materials.
