# Supplementary material for: Chemical Constituents of Eupatorium japonicum and Anti-Inflammatory, Cytotoxic, and Apoptotic Activities of Eupatoriopicrin on Cancer Stem Cells
Source: Evid Based Complement Alternat Med. 2021 May 18;2021:6610347. doi: 10.1155/2021/6610347 (PMC8149239; doi:10.1155/2021/6610347)
Supplement: Supplementary Materials — Experimental details and MS and NMR spectra of compounds 1–17 are freely available along with the manuscript as supplementary materials. [file 6610347.f1.doc]

**SUPPLEMENTARY MATERIALS**

**Chemical constituents of *Eupatorium japonicum* and anti-inflammatory, cytotoxic/apoptotic activities of eupatoriopicrin on cancer stem cells**

Minh Giang Phan,^a,*^ Thi Thao Do,^b^ Thi Nga Nguyen,^b^ Thi Viet Huong Do,^a^ Ngoc Phuc Dong,^a^ Minh Trang Vu ^c^

*^a^Faculty of Chemistry, VNU University of Science, Vietnam National University, Hanoi, 19 Le Thanh Tong Street, Hanoi, Vietnam*

*^b^Institute of Biotechnology, Vietnam Academy of Science and Technology, 18 Hoang Quoc Viet Road, Hanoi, Vietnam*

*^c^VNU University of Education, Vietnam National University, Hanoi, 144 Xuan Thuy Road, Hanoi, Vietnam*

*** Corresponding Author. *E-mail*: giangpm@vnu.edu.vn (M. G. Phan)

**Abstract**

*Eupatorium japonicum* Thunb. of the plant family Asteraceae is a popular traditional herb in Vietnam. However, its chemical constituents as well as the bioactive principles have not been investigated yet. We investigated the phytochemistry of *E. japonicum* in Vietnam and isolated seventeen compounds (**1**-**17**) including phytosterols, terpenoids, phenolic acids, flavonoids, fatty alcohols, and fatty acids. They were structurally determined by MS and NMR analysis. Except compounds **6** and **12** all the other compounds were identified for the first time from *E. japonicum*. Since many sesquiterpene lactones with *α*-methylene *γ*-lactone ring are reported as anti-inflammatory and anticancer agents eupatoriopicin (**10**) and 1-hydroxy-8-(4,5-dihydroxytigloyloxy)eudesma-4(15),11(13)-dien-6,12-olide (**11**) were selected among the isolates for biological assays. Compound **10** was identified as the main bioactive sesquiterpene lactone of *E. japonicum* showing its potent anti-inflammatory and cytotoxic activity through inhibiting NO production and the growth of HepG2 and MCF-7 human cancer cell lines. For the first time eupatoriopicrin (**10**) was demonstrated to strongly inhibit NTERA-2 human cancer stem cell line *in vitro*. It is noticeable that the cytotoxicity of eupatoriopicrin against NTERA-2 cells is mediated by its apoptosis-inducing capability of **10** as demonstrated by the results of Hoechst 33342 staining, flow cytometry apoptosis analysis, and caspase-3 activity assays. The biological activities of the main bioactive constituents **1**-**7**, **10**, **12**, and **15** supported the reported anti-inflammatory and anticancer properties of extracts from *E. japonicum*.

**Keywords:** *Eupatorium japonicum*, sesquiterpene lactone, eupatoriopicrin, cytotoxicity, NO production, apoptosis, cancer stem cell.

**Legend**

**Figure S1.** ^1^H-NMR (CDCl_3_) of compounds **1/2**

**Figure S2.** ^1^H-NMR (CDCl_3_) of compounds **3**/**4**

**Figure S3.** ^1^H-NMR (CDCl_3_) of compounds **5**/**6**

**Figure S4.** IR of compound **5**

**Figure S5.** ^1^H-NMR (CDCl_3_ + CD_3_OD) of compound **7**

**Figure S6.** ^1^H-NMR (CD_3_OD) of compound **8**

**Figure S7.** ^1^H-NMR (CDCl_3_) of compound **9**

**Figure S8.** ^1^H-NMR (CD_3_OD) of compound **9**

**Figure S9.** ^1^H-NMR (CDCl_3_) of compound **10**

**Figure S10.** ^13^C-NMR (CDCl_3_) of compound **10**

**Figure S11.** DEPT (CDCl_3_) of compound **10**

**Figure S12.** Positive-ion ESI-MS of compound **10**

**Figure S13.** ^1^H-NMR (CD_3_OD) of compound **10**

**Figure S14.** ^13^C-NMR (CD_3_OD) of compound **10**

**Figure S15.** DEPT (CD_3_OD) of compound **10**

**Figure S16.** ^1^H-NMR (CDCl_3_) of compound **11**

**Figure S17.** ^13^C-NMR (CDCl_3_) of compound **11**

**Figure S18.** DEPT (CDCl_3_) of compound **11**

**Figure S19.** Positive-ion ESI-MS of compound **11**

**Figure S20.** Negative-ion ESI-MS of compound **11**

**Figure S21.** ^1^H-NMR (CD_3_OD) of compound **12**

**Figure S22.** ^1^H-NMR (CD_3_OD) of compound **13**

**Figure S23.** ^13^C-NMR (CD_3_OD) of compound **13**

**Figure S24.** DEPT (CD_3_OD) of compound **13**

**Figure S25.** ^1^H-NMR (CD_3_OD) of compound **14**

**Figure S26.** ^13^C-NMR (CD_3_OD) of compound **14**

**Figure S27.** DEPT (CD_3_OD) of compound **14**

**Figure S28.** ^1^H-NMR (CD_3_OD) of compound **15**

**Figure S29.** ^13^C-NMR (CD_3_OD) of compound **15**

**Figure S30.** ^1^H-NMR (CD_3_OD) of compound **16**

**Figure S31.** ^13^C-NMR (CD_3_OD) of compound **16**

**Figure S32.** DEPT (CD_3_OD) of compound **16**

**Figure S33.** ^1^H-NMR (CD_3_OD) of compound **17**

**Figure S1.** ^1^H-NMR (CDCl_3_) of compounds **1**/**2**

**Figure S2.** ^1^H-NMR (CDCl_3_) of compounds **3**/**4**

**Figure S3.** ^1^H-NMR (CDCl_3_) of compounds **5**/**6**


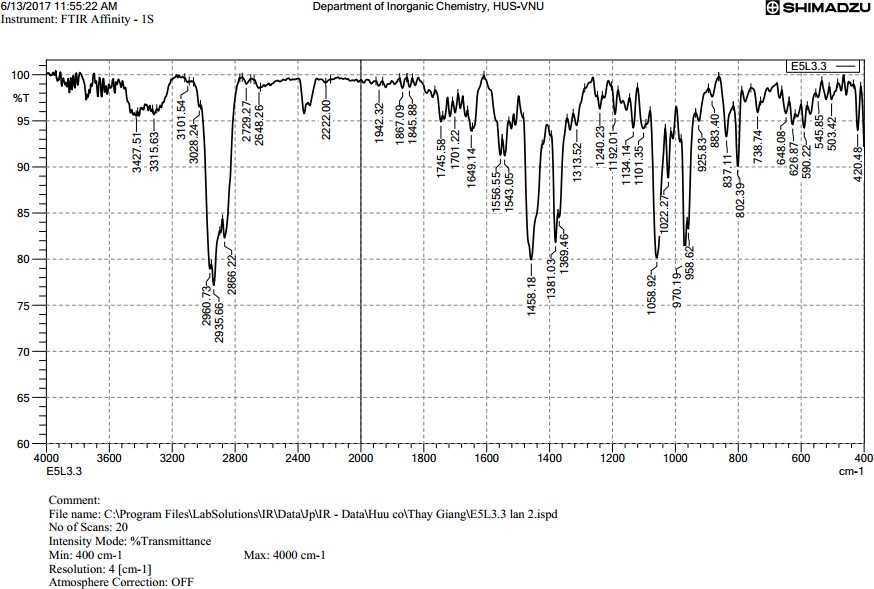


**Figure S4.** IR of compound **5**

**Figure S5.** ^1^H-NMR (CDCl_3_+CD_3_OD) of compound **7**

**Figure S6.** ^1^H-NMR (CD_3_OD) of compound **8**

**Figure S7.** ^1^H-NMR (CDCl_3_) of compound **9**

**Figure S8.** ^1^H-NMR (CD_3_OD) of compound **9**

**Figure S9.** ^1^H-NMR (CDCl_3_) of compound **10**

**Figure S10.** ^13^C-NMR (CDCl_3_) of compound **10**

**Figure S11.** DEPT (CDCl_3_) of compound **10**

**Figure S12.** Positive-ion ESI-MS of compound **10**

**Figure S13.** ^1^H-NMR (CD_3_OD) of compound **10**

**Figure S14.** ^13^C-NMR (CD_3_OD) of compound **10**

**Figure S15.** DEPT (CD_3_OD) of compound **10**

**Figure S16.** ^1^H-NMR (CDCl_3_) of compound **11**

**Figure S17.** ^13^C-NMR (CDCl_3_) of compound **11**

**Figure S18.** DEPT (CDCl_3_) of compound **11**

**Figure S19.** Positive-ion ESI-MS of compound **11**

**Figure S20.** Negative-ion ESI-MS of compound **11**

**Figure S21.** ^1^H-NMR (CD_3_OD) of compound **12**

**Figure S22.** ^1^H-NMR (CD_3_OD) of compound **13**

**Figure S23.** ^13^C-NMR (CD_3_OD) of compound **13**

**Figure S24.** DEPT (CD_3_OD) of compound **13**

**Figure S25.** ^1^H-NMR (CD_3_OD) of compound **14**

**Figure S26.** ^13^C-NMR (CD_3_OD) of compound **14**

**Figure S27.** DEPT (CD_3_OD) of compound **14**

**Figure S28.** ^1^H-NMR (CD_3_OD) of compound **15**

**Figure S29.** ^13^C-NMR (CD_3_OD) of compound **15**

**Figure S30.** ^1^H-NMR (CD_3_OD) of compound **16**

**Figure S31.** ^13^C-NMR (CD_3_OD) of compound **16**

**Figure S32.** DEPT (CD_3_OD) of compound **16**

**Figure S33.** ^1^H-NMR (CD_3_OD) of compound **17**
